# Prioritizing outcome measures after aneurysmal subarachnoid hemorrhage: A q-sort survey of patients, health care providers and researchers

**DOI:** 10.3389/fneur.2022.1068499

**Published:** 2022-11-25

**Authors:** Christopher R. Andersen, Justin Presseau, Victoria Saigle, Emily Fitzgerald, Madeline Lamanna, Phil Talbot, Anthony Delaney, Shane W. English

**Affiliations:** ^1^Northern Clinical School, Sydney University, Sydney, NSW, Australia; ^2^The George Institute for Global Health, UNSW, Newtown, NSW, Australia; ^3^Intensive Care Department, Royal North Shore Hospital, St Leonards, NSW, Australia; ^4^School of Epidemiology and Public Health, University of Ottawa, Ottawa, ON, Canada; ^5^Ottawa Hospital Research Institute (OHRI), Ottawa, ON, Canada; ^6^Independent Researcher, Sydney, NSW, Australia; ^7^Department of Medicine (Critical Care), University of Ottawa, Ottawa, ON, Canada

**Keywords:** subarachnoid hemorrhage, core outcome set (COS), patient reported outcome measure (PROM), Q-method analysis, outcome assessment (health care), stroke

## Abstract

**Objective:**

To understand which outcome measures patients and their families, health care providers, and researchers prioritize after aneurysmal subarachnoid hemorrhage (aSAH).

**Methods:**

We conducted a cross-sectional q-sort survey with participants from three key stakeholder groups. Potential outcomes were identified from interviews and focus groups. Participants were purposively sampled to achieve diversity based on stakeholder group, geography, and profession. Respondents sorted 27 outcomes in a quasi-normally distributed grid (Q-Sort) from most to least important. Principal components analysis was used to determine similarities in the way participants sorted the outcome measures resulting in distinct groupings. Overall rankings were also reported.

**Results:**

112 participants were invited. 70 responded and 64 participants from 25 different countries completed a Q-sort. Balanced stakeholder representation was achieved. Five distinct patterns were identified based on survival, pathophysiological, psychological, resource use, and functional outcome measures. Quality of life as reported by the patient was the highest ranked outcome measure followed by independence and functional measures. Survival and biomedical outcomes were ranked in the middle and cost measures last.

**Conclusions:**

In this diverse sample of key stakeholders, we characterized several distinct perspectives with respect to outcome measure selection in aSAH. We did not identify a clear pattern of opinion based on stakeholder group or other participant characteristics. Patient-reported measure of quality of life was ranked the most important overall with function and independence also highly rated. These results will assist study design and inform efforts to improve outcome selection in aSAH research.

## Introduction

Clinical trials define an “outcome” as a measurement or observation used to assess the effect of an intervention or process of care with respect to side effects (risks) and effectiveness (benefits) (1). Outcome selection is a complex task that requires consideration of multiple factors including the research question, target population, intervention, and comparator being studied. Also important are the measurement properties of the available instruments, costs, ease of data collection and burden on participants (2). Selecting outcomes that are relevant to the end-users of research: patients, health care providers and policy makers is crucial. High quality research can assist these stakeholders in decision making and ensure the best care is delivered (3). There is clear evidence that the choice of outcome measures by researchers often does not align with the priorities of the end-users (4, 5). When this mismatch occurs researchers risk designing studies that overlook key factors and could interpret some interventions to be effective when they are not (and at worst cause harm) (6).

Aneurysmal subarachnoid hemorrhage (aSAH) is a devastating type of stroke triggered by the sudden rupture of an abnormal blood vessel in the brain. It affects younger patients when compared to other forms of stroke, has a distinct clinical course, and survivors are often left with long term impairment (7, 8). Our previous work has demonstrated that there is high degree of heterogeneity in the outcome measures used in aSAH research (9). We have also shown that the perspectives of patients and families members in the development of outcome measures are often overlooked (10). Additionally, there is evidence of a failure to meet the needs of aSAH survivors especially with respect to poorly-reported outcomes such as fatigue, mood and cognition (11).

Our objective is to characterize the perspectives of patients, researchers and health care providers and understand which outcomes measures in aSAH are considered the highest priority. We also aim to identify if there are clearly different viewpoints between our three stakeholder groups with respect to outcome prioritization. This work is designed to inform efforts to standardize outcome selection and ensure the selected outcomes in aSAH research align with priorities of research end users (12).

## Methods

### Standard protocol approvals, registrations, and patient consents

Ethical approval for the survey development work was provided by the Ottawa Health Science Network Research Ethics Board (Reference 20190312-01H) and by Northern Sydney Local Health District Reference: 2020/ETH03188 for the q-sort recruitment and administration. Reporting of this study has been conducted consistent with the Checklist for Reporting of Survey Studies (CROSS) (13).

### Q methodology

Q-methodology is a valuable technique for exploring subjective opinion with respect to priorities in healthcare. It can be used to identify groups of participants who have shared viewpoints or make sense of a pool of comparable items in similar ways (14). Importantly, it requires participants to differentially value a collection of statements (in this case outcome measures) by ranking them between two extremes such as most important to least important. This ranking process is called q-sorting. The resultant rankings are then analyzed through factor analysis to identify similar viewpoints.

**Figure 1:**
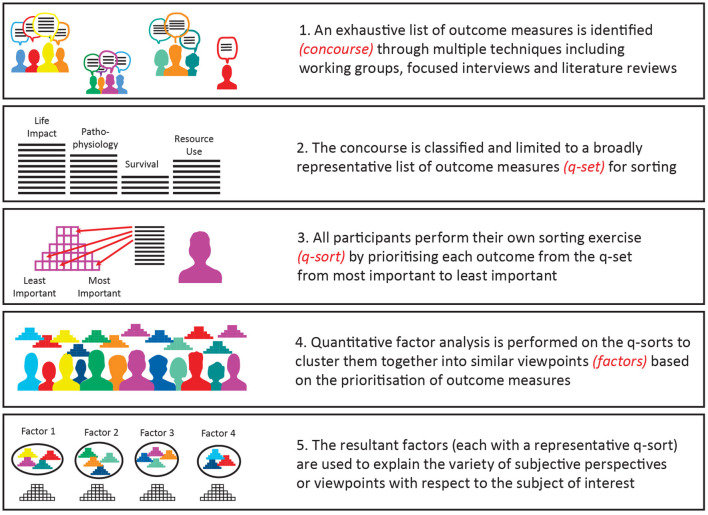
Overview of Q-methodology.

This process is described in detail in Figure 1. The first step is to create a concourse of statements through a variety of techniques and achieve as broad a range of statements as possible. The concourse is then reduced by the researchers into a manageable but representative list (q-set) that can be used for sorting. The participants are provided with the q-set and then rank the statements between two extremes. The pattern for the distribution is usually a quasi-normally distributed grid, although other patterns are also effective. A quantitative analysis is then performed on the q-sorts to identify shared perspectives or viewpoints called factors. There is a representative q-sort for each factor that enables qualitative interpretation of the results (15–17).

### Survey development

The concourse was developed through several techniques including a working group with members of all three stakeholder groups at the International Subarachnoid Hemorrhage Conference in Amsterdam in June 2019 (18). Additionally, we ran a focus session with aSAH survivors and their families in North America, and interviews with health care providers and aSAH survivors in the UK and Australia. A final concourse of 106 outcome measures was identified (Appendix 1).

The concourse classified into 4 core areas (pathophysiological, life impact, death and resource use) consistent with the OMERACT Filter for outcome classification in clinical research (19). It has been recommended that an outcome measure from each of these core areas is included in core outcome sets (COS) used to improve the consistency of outcome measure selection (20). The research team, including patient research partners, then reduced the overall concourse to 27 items by consensus for a final q-set (Appendix 1). We used a quasi-normal grid for our q-sort with a −4 to +4 distribution (21).

We piloted the survey with members of each stakeholder group who were also part of our larger research group for clarity and ease of completion. Based on feedback, we refined the participant instructions and provided additional links for assistance.

### Recruitment

In accordance with recommendations of the q-method literature, a sample size of 60 was chosen (14). Participants were recruited *via* email (Appendix 2) who had previously registered interest in improving outcome selection after being approached *via* patient, research and healthcare provider organizations. A purposive sampling technique was employed to ensure broad representation based on personal or professional involvement with aSAH, self-identified gender, and geographical location (14). All communication between the researchers and the participants was *via* email.

### Survey administration

Invited participants were provided a randomly generated alphanumeric code and link to complete the survey to prevent multiple participations. Participants conducted the q-sort using Q-method software online platform (22). After providing informed consent and completing the demographic questions, participants were given a text explanation (Appendix 2) and video explanation (23) on how to complete a q-sort. The outcome measures in the q-set were presented randomly to participants. They were asked to prioritize the outcomes with the prompt “When we are testing a new treatment(s) for subarachnoid hemorrhage, in your personal opinion do you think measuring this domain, outcome or indicator of health is more important, neutral or less important when compared to the others in this list”. After sorting the outcomes into 3 categories the participant progressed to placing them on the final q-sort according to their priorities. At the completion of the q-sort, 4 optional questions were provided for participants to explain their rationale for their preferences, whether there were any missing outcomes in the q-set and their experience completing the survey. Data were stored on password protected University of Sydney servers and responses were associated with the unique code rather than identifiable information to ensure confidentiality.

### Survey analysis

A quantitative analysis of the overall configurations decided by participants was conducted using Q Method Software (22). Only completed q-sorts were included in the analysis. The extraction method was performed *via* principal components analysis and eigenvalues >1 was used to determine factors to rotate. Significance loadings were set at *p* < 0.01. The rotation method was varimax. The method of factor flagging was automatic. These methods are the most commonly used in health care settings (21, 24, 25). The variance explained by each factor was calculated by the formula (eigenvalue times number of participants/100) (26). Extracted factors that explained at least 5% of the overall variance were reported (27). The reliability and standard error of the z-scores were also reported (28). There was no weighting or propensity matching performed. Additionally, an exploratory analysis of the overall outcome measure rankings across all participants was performed. The ideal q-sorts that represent each of the factors are described qualitatively following the methods described by Watts and Stenner (27).

## Results

**Figure 2:**
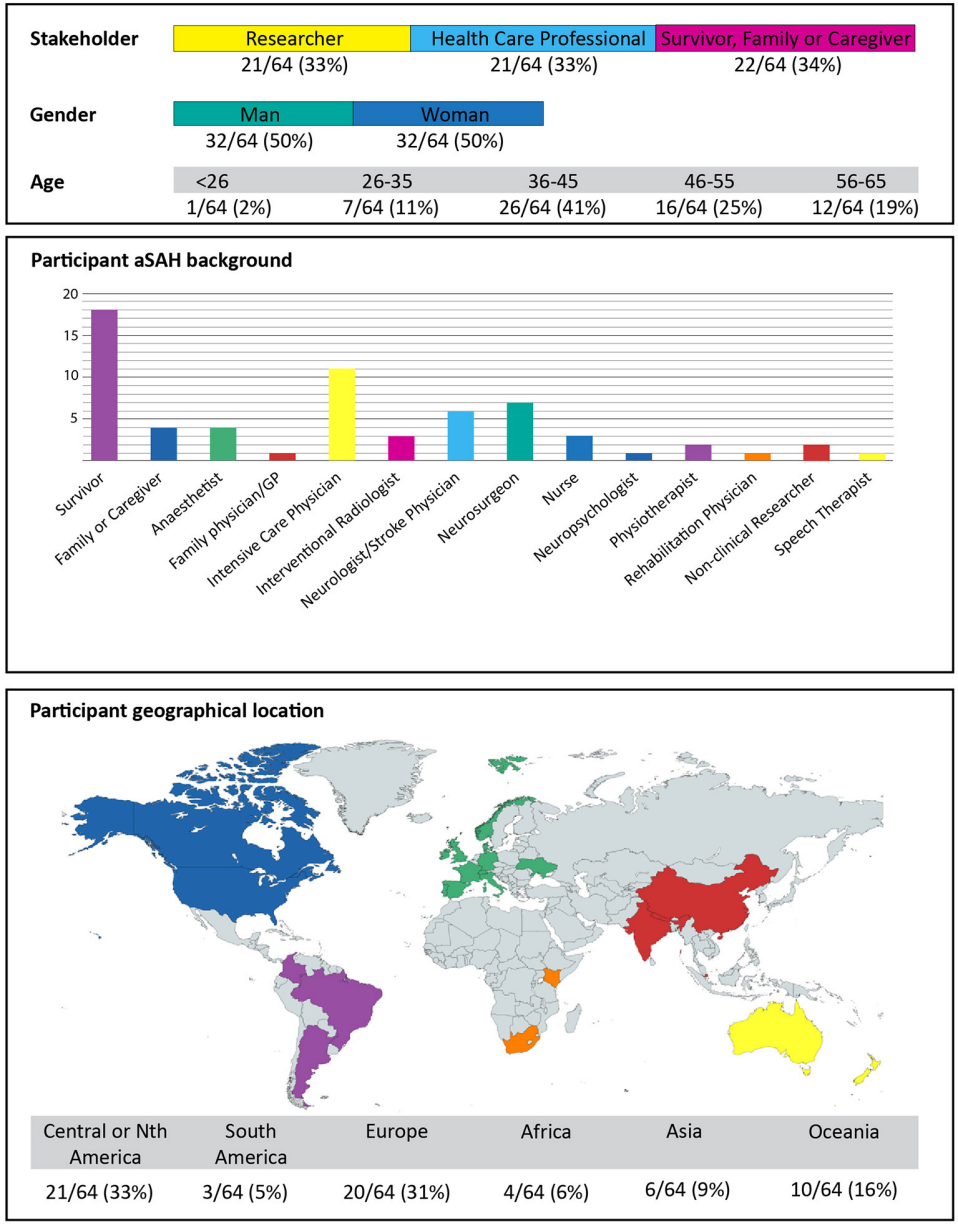
Participant demographics.

From 112 emailed invitations to take part there were 70 responses (63%) with 64 q-sorts completed (91%) over a two-month period from 3rd September 2021. Baseline demographic details are provided in Figure 2.

In the exploratory analysis the highest ranked outcome measure across all respondents was the overall quality of life (QoL) as reported by the aSAH survivor (Table 1). Independence in basic needs and instrumental activities, as well as a measure of function or return to baseline function all ranked in the top 5. The highest ranked pathophysiological outcome was delayed cerebral ischemia (a common complication after aSAH that is associated with worse outcomes) which ranked 4th out of 27. Measures of death included being alive 3 months after the aSAH and being discharged from hospital alive ranked 8th and 15th, respectively. Outcome measures of the cost of the initial hospital admission and the cost of rehabilitation and treatment after hospital discharge were rated 26th and 27th out of 27.

**Table 1:** Overall outcome measure rankings from all participants.

**Rank**	**Outcome measure**	**Overall rating**
1	The overall quality of life as reported by the SAH survivor	99
2	The ability to independently manage basic needs such as toileting, feeding, bathing and getting dressed	97
3	A measure of function or a return to baseline function	62
=4	Delayed cerebral ischemia or cerebral infarction (a common complication in the days after SAH that is associated with worse outcomes)	57
=4∧	The ability to independently manage instrumental activities of daily living such as managing finances, shopping, preparing food, and doing laundry	57
6	An assessment of memory and cognitive function	49
7	The ability to walk independently	47
8	Being alive (survival) three months after the subarachnoid hemorrhage	44
=9	A subsequent bleed related to the aneurysm (rebleeding)	38
=9∧	Being able to return to work	38
11	The ability to speak fluently	32
12	The ability to maintain concentration and focus	20
13	Feelings of anxiety and/or symptoms of post-traumatic stress disorder	11
14	A measure of the overall impact on family and caregivers	7
15	Being discharged from hospital alive	6
16	Symptoms of depression and/or a more general assessment of mood	3
17	The frequency and severity of pain related to the SAH including headaches	−2
18	Vasospasm (the narrowing of arteries) in the first days and weeks after SAH	−17
19	Overall energy levels and how easy it is to fatigue	−29
20	The destination after discharge from hospital (for example home, a rehabilitation facility or a residential care facility)	−52
21	The overall speed of recovery	−65
22	The ability to attend social functions such as dinners, birthdays and other gatherings	−66
23	A return to normal sexual activity and function	−68
24	The ability to return to driving	−69
25	The length of stay in intensive care or hospital	−75
=26	The overall cost of the initial hospital admission	−112
=26∧	The overall cost of rehabilitation and treatment after hospital discharge	−112

### Factor descriptions

**Figure 3:**
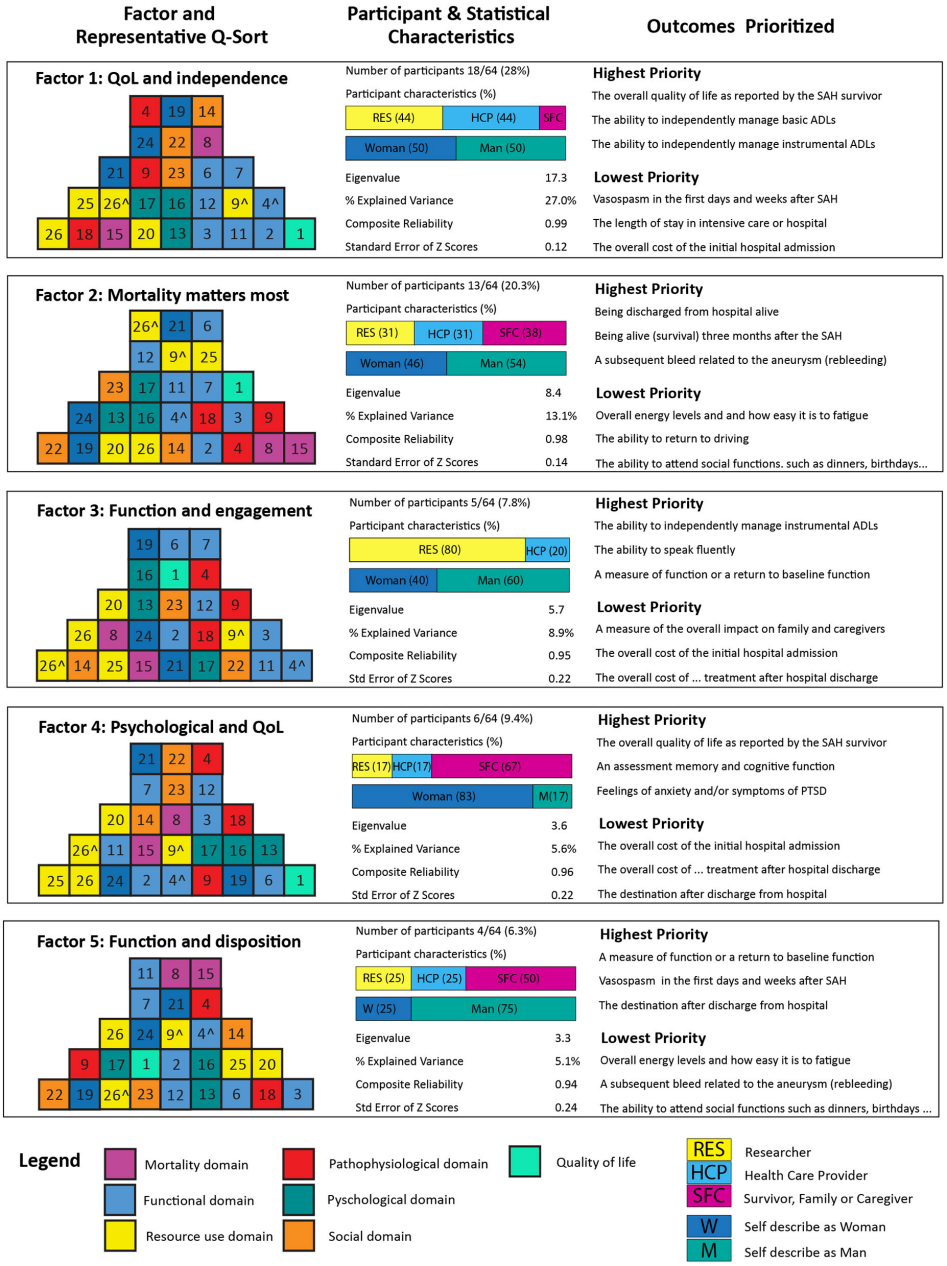
The representative q-sorts (colored pyramids) for each of the 5 identified factors are shown. The highest ranked outcome measure is in the right corner, descending in importance as you move to the left of the q-sort. In each pyramid column the higher ranked outcome measure is at the base. The numbers for each outcome measure correspond to the overall rankings in Table 1. The number of participants loading to each factor, the demographic characteristics of these participants and the statistical characteristics are shown. In descending order, the three highest and lowest ranked outcome measures is also presented (full outcome measure rankings for each of the 5 factors is presented in the Supplemental material).

Five distinct viewpoints (factors) were identified by the factor analysis, with almost half of all the participants (31/64) loading to either Factor 1 or Factor 2. The individual factors are described below with a summary of each factor provided in Figure 3. Ten participants loaded to multiple factors and eight participants loaded to factors that explained <5% of the total variance. The full 27-item prioritization for each factor is provided in Appendix 3.

### Factor 1: Patient reported outcomes are best

This was the most common viewpoint overall and explained over a quarter of the total sample variance (see Figure 3). This viewpoint was shared by participants from each stakeholder group however researchers and healthcare providers predominate. The most characteristic outcome measures for this group were quality of life and independence. Almost all positively scored outcome measures in this viewpoint were related to how a patient feels, functions, or survives. Pathophysiological outcome measures including rebleeding, vasospasm and delayed cerebral were all negatively rated with cost viewed as least important. A reoccurring theme in the post q-sort statements was the primacy of patient reported outcome measures “Patient reported outcomes are best as they reflect what is important to the person that has had the aSAH” and quality of life “The most important outcomes center around the patient being able to maintain quality of life”.

### Factor 2: Mortality matters most

People from this viewpoint placed measures of survival in the two highest positions on the representative q-sort. Additionally, they also prioritized pathophysiological outcomes when compared to functional, psychological or measures of independence. When compared to the other perspectives this difference was marked. The other notable characteristic was cost rating in the mid-range when compared to most other perspectives that considered these outcomes least important. The post p-sort statements included the rationale for focusing on survival such as “As a neurosurgeon I am biased to protect the patient's life first”.

### Factor 3: Function and social engagement over survival

People from this viewpoint prioritized instrumental activities, and placed outcomes related to social interaction such as the ability to speak fluently, attend social functions and return to work notably higher than other viewpoints. This contrasted with survival which this factor placed notably lower than all other viewpoints. There were no stakeholders from the survivor, family and caregiver group that loaded to this factor. Statements included comments on ‘Returning to one's prior function, activity and to be as symptom free as possible to be the most important”.

### Factor 4: Psychological outcomes, fatigue, and quality of life

This was the only factor where most participants were from the patient, family, and caregiver stakeholder group. Like factor 1, patient reported QoL was rated highest. This factor was notable however in the priority placed on psychological outcomes that included “feelings of anxiety and/or symptoms of post-traumatic stress disorder” and “symptoms of depression and/or a general assessment of mood”. Participants also placed a much higher priority on fatigue when compared to the other factors. This was further reflected in the post q-sort statements that suggested there should be a focus on “neurofatigue, depression, anxiety and how there is an adjustment to one's previous way of functioning…” and another participant commented that “For me, the severe PTSD, depression, pain, insomnia, not knowing what was normal…” was important. A third participant made the statement “cognitive and emotional problems with clinical significance, with fatigue one of the most prominent symptoms”.

### Factor 5: Function, discharge destination, and length of stay

This viewpoint prioritized functional outcomes but unlike factor 3 the social engagement outcomes were not seen as important with the ability to attend social functions considered least important, as well as patient reported QoL, language fluency and overall energy levels and how easy it is to fatigue all negatively rated. Respondents in factor 5 also prioritized more outcome measures related to patient disposition (such discharge destination and length of stay in hospital/ICU) much higher when compared to other viewpoints. Respondents commented on “are you able to live in your own home? What kind of support is available post-acute hospitalization”?

## Discussion

Our novel study has demonstrated that there are several distinct perspectives on the ideal outcome measures after aSAH. Overall, the respondents from this study identified patient-reported quality of life as the most important outcome measure. Measures of independence and functional outcome measures were also highly rated. Measures of survival and psychological outcomes are very important to some even if this is not reflected in the overall rankings. Indicators of resource use were generally not prioritized relative to the other outcome measures in this study.

These results support concerns that researchers in the field are not currently measuring what matters most to relevant stakeholders. Although seen as the most important outcome to respondents in this survey, patient-reported outcome measures (either as a primary or secondary outcome) have been reported in only 8.5% of randomized controlled trials in aSAH over the past 20 years (9). Psychological outcomes are also very important to some respondents but are rarely reported, which should be considered when selecting outcome measures for future studies. This research does however support measuring functional and independence outcomes that are commonly reported in aSAH research. We anticipate the results of this study will aid researchers to make more informed decisions when selecting outcome measures to test the effectiveness of future interventions.

One of the strengths of this study is the broad sample that has been achieved through purposive sampling. Participants were recruited from 25 different countries across all geographical regions representing many different health care and socio-economic settings. The recruited participants also included patients, family members as well as health care providers who are involved across all aspects of the patient experience from the acute admission through rehabilitation and care in the community. This increases the chances that we have captured a very wide range of perspectives. Our research team involved patient research partners (PT), non-clinical researchers (JP, ML and VS) and clinician researchers (CA, EF, SE, and AD) ensuring key stakeholder involvement throughout entire study process from design to write up. The use of a rigorous technique such as q-methodology also allowed us to use quantitative techniques to identify similarities of perspective in a robust way without the influence of prior assumptions.

The weakness of this study is that although we have a range of participants the purposive sampling means that we cannot assume that this is a representative sample. Efforts were made to ensure participation from not just high-income countries, but we may not have achieved equal representation of these groups. These concerns may not influence the factor analysis but there should be caution with the exploratory analysis on the overall rankings. Further investigation using more traditional quantitative analyses are required to confirm the generalizability of the overall rankings and increased representation for the survivors, family and caregiver group for this type of analysis would be prudent.

There is marked heterogeneity in outcome measures used to evaluate treatments of aSAH (9). The lack of consistent outcome measures in this area hinders comparison of trials and reduces the utility of research. The results of this study will be used to inform efforts to improve outcome measure selection in aSAH. An international consortium including patients, health care providers, journal editors, foundation representatives and leading researchers recently proposed the development of a core outcome set (COS) in SAH to address the limitations of current aSAH outcome measure selection (18). A COS is a limited set of agreed outcome measures which all studies of a particular area of medicine will report (19). Central to developing a COS is engaging the key stakeholders and ensuring consideration of a range of different viewpoints as explored in this study. Other work including an international modified Delphi study and consensus meeting will help to finalize a COS in aSAH is currently in progress.

## Conclusions

We have demonstrated that there are several distinct perspectives or viewpoints with respect to outcome measure selection after aSAH. Most perspectives rated patient-reported quality of life highly or of the highest priority despite this being rarely reported in the literature. There is general support for measuring function and independence. Survival and psychological outcomes appear very important to specific groups, but this nuance may be lost when looking at overall rankings. Understanding and incorporating these perspectives when selecting outcome measures is crucial for ensuring we drive improvements in aSAH management that matter to all stakeholders.

## Data availability statement

The datasets generated or analyzed during the current study are available from the corresponding author on reasonable request and may require interinstitutional data-sharing agreements to be put in place.

## Ethics statement

Ethical approval for the survey development work was provided by the Ottawa Health Science Network Research Ethics Board (Reference 20190312-01H) and by Northern Sydney Local Health District Reference: 2020/ETH03188 for the q-sort recruitment and administration. The patients/participants provided their written informed consent to participate in this study.

## Author contributions

CA designed and conceptualized the study, conducted recruitment, analyzed the data, and drafted and revised the manuscript for intellectual content. SE, JP, PT, and AD designed and conceptualized the study and revised the manuscript for intellectual content. VS, ML, and EF assisted with survey development and revised the manuscript for intellectual content. All authors contributed to the article and approved the submitted version.

## Funding

This work was supported by The Ottawa Hospital Academic Medical Organization Innovation Fund Grant provided to improve outcome measurement in subarachnoid hemorrhage.

## Conflict of interest

The authors declare that the research was conducted in the absence of any commercial or financial relationships that could be construed as a potential conflict of interest.

## Publisher's note

All claims expressed in this article are solely those of the authors and do not necessarily represent those of their affiliated organizations, or those of the publisher, the editors and the reviewers. Any product that may be evaluated in this article, or claim that may be made by its manufacturer, is not guaranteed or endorsed by the publisher.
