# Supplementary material for: Prioritizing outcome measures after aneurysmal subarachnoid hemorrhage: A q-sort survey of patients, health care providers and researchers
Source: Front Neurol. 2022 Nov 25;13:1068499. doi: 10.3389/fneur.2022.1068499 (PMC9732721; doi:10.3389/fneur.2022.1068499)
Supplement: Supplementary file 1 [file Data_Sheet_1.PDF]

Supplemental Information

Appendix 1 – Concourse and Q-Set

Pathophysiological	Life Impact	Resource Use	Death
Aneurysm obliteration	Ability to attend social functions	Access to rehab	Survival to hospital discharge
Anxiety	Ability to multi-task	Availability and access to therapy	Survival at 3 months
Aphasia	Ability to respond to emergencies	Disposition - Discharge	Survival at 1 month
Backpain	Ability to take care of self	Financial impact (family/society)	Survival after surgery
Balance	Ability to work/Return to work/Struggling with work	Hospital cost	
Cerebral infarction	Ability to write legibly	Hospital free days	
Cognition	Able to live alone	Hospital staff	
Concentration/Focus	ADLs	ICU free days	
CSF Flow	Caregiver - Knowledge on how to support person with aneurysm	Length of stay - Hospital	
Decision-making ability (e.g. speed)	Caregiver health	Length of stay - ICU	
Depression	Caregiver Impact	Length of stay - Rehabilitation	
Difficulty with Noise	Caregiver PTSD/burnout/depression	MRIs (for new/existing aneurysms)	
Early brain injury (CT, MRI, serum/CSF biomarker)	Cooking	Occupational therapy	
Emotions	Driving	Ongoing care cost	
Ensuring other potential health issues aren't missed (e.g. unrelated to aneurysm)	Eating independence	Permanent institutionalisation	
Executive Function	Family - Emotional support	Quality of Care	
Fatigue/energy level	Family impact	Rehabilitation resource use	
Frustration	Functional outcome	Speech therapy	
Headache	Giving up coffee	Therapeutic Intensity	
ICU Complications	Household chores (cleaning, yardwork, laundry)	Use of medications	
Incidence of vasospasm	IADLs		
Lack of taste/smell	Information for family		
Less emotional	Isolation		
Memory	Micrographia		
Mood	Peer support/Connection with other SAH survivors		
More emotional	Quality of Life		
Neurofunctional disability	Return to baseline		
Neuropsych	Return to previous role		
New neuro-deterioration secondary to DCI, etc.	Shopping		
Olfactory Function	Social stigma of having had brain surgery		
Physical health of person with aneurysm	Spelling		
Pituitary Function	Support from family		
PTSD	Taking care of children		
Rebleeding	Understanding what is happening/what will happen		
Recanulization	Walking/walking without cane		
Retreatment			
Seizure			
Sensory overload			
Sexual function/activity			
Shunt Dependency			
Sleep			
Speech/word-finding			
Speed of recovery			
Substance Use			
Tracheotomy Requirement			
Vestibular Issues			
Visual Function			

eTable A: Concourse of abbreviated statements and outcomes from workshops, focus groups and interviews

Statement Number	Statements
**1**	Feelings of anxiety and/or symptoms of post traumatic stress disorder
**2**	Symptoms of depression and/or a more general assessment of mood
**3**	The ability to maintain concentration and focus
**4**	Overall energy levels and how easy it is to fatigue
**5**	The frequency and severity of pain related to the SAH including headaches
**6**	A return to normal sexual activity and function
**7**	The ability to speak fluently
**8**	Delayed cerebral ischemia or cerebral infarction (a common complication in the days after SAH that is associated with worse outcomes)
**9**	Vasospasm (the narrowing of arteries) in the first days and weeks after SAH
**10**	The overall speed of recovery
**11**	A subsequent bleed related to the aneurysm (rebleeding)
**12**	The ability to attend social functions such as dinners, birthdays and other gatherings
**13**	The ability to independently manage basic needs such as toileting, feeding, bathing and getting dressed
**14**	The ability to independently manage instrumental activities of daily living such as managing finances, shopping, preparing food, and doing laundry
**15**	Being able to return to work
**16**	A measure of the overall impact on family and caregivers
**17**	The ability to return to driving
**18**	A measure of function or a return to baseline function
**19**	The ability to walk independently
**20**	The destination after discharge from hospital (for example home, a rehabilitation facility or a residential care facility)
**21**	The overall cost of the initial hospital admission
**22**	The overall cost of rehabilitation and treatment after hospital discharge
**23**	The length of stay in intensive care or hospital
**24**	Being discharged from hospital alive
**25**	Being alive (survival) three months after the subarachnoid haemorrhage
**26**	The overall quality of life as reported by the SAH survivor
**27**	An assessment memory and cognitive function

eTable B. Final Q-Set of 27 Items

Appendix 2 – Survey details and information provided to participants

Invitation email

Dear XXX,

Thank you for demonstrating your interest in participating in our work to identify which outcomes or health indicators after subarachnoid haemorrhage (SAH) matter the most to patients and families, health care workers and researchers.

In previous in-depth interviews and working groups with our three stakeholders we collected 27 different outcomes or health indicators. We would like you to sort these outcomes or health indicators from what you think is most important (on the right) through to least important (on the left). This takes most people around 15-20 minutes to complete but there is no time limit.

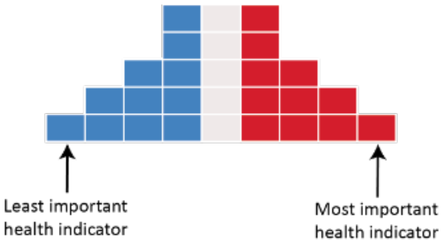

Your link to undertake the survey is here [unique hyperlink provided]

We have also attached the participant information sheet as required by our Human Research Ethics Committee. Please feel free to reply to this email if you have any questions with this novel survey, your opinion is very important in guiding this project and we are very keen to assist! Also, if you are unable to take part currently, we understand what a challenging time it is and thank you for considering - we would only ask that you send a quick reply to this email so we can find a suitable replacement.

Many thanks again,

Q-Sort Details (after using hyperlink in email)

Survey Consent Process

You are invited to take part in this research project, which is called Measuring Attitudes to Health Indicators Post Subarachnoid Haemorrhage (MARIPoSA). You have been invited because we are interested in understanding your personal opinions about subarachnoid haemorrhage. You should have received a detailed patient information sheet when you were emailed the link to this survey and you are encouraged to read this information and ask any questions that you may have before taking part.

Participation in this research is voluntary. If you don't wish to take part, you don't have to.

If you decide you want to take part in the research project, you will be asked to indicate consent via the consent section. Clicking on the consent button you are telling us that you:

Understand what you have read

- Consent to take part in the research project
- Consent to be involved in the research described
- Consent to the use of your personal and health information as described.

It will take most participants around 15-20 minutes to complete the q-sort exercise.

Initial instructions to participants

Please sort the initial set of statements into three different categories ranging from what you consider most important, neutral and less important. There is no need to sort statements equally into the three categories although placing a few into the most important and less important and the rest into neutral will make the next step easier.

Once you have commenced an initial sort you can continue on and complete the final sorting exercise. This will place each of the statements into the pyramid from most important (to the right of the pyramid) to less important (on the left of the pyramid). Most statements will be in the middle of the pyramid. You are free to change your mind from the initial sort and place statements anywhere on the pyramid, as well as change the positions around up until you have decided that the distribution is correct in your opinion. You can zoom in and out to help with reading the text which is often quite small.

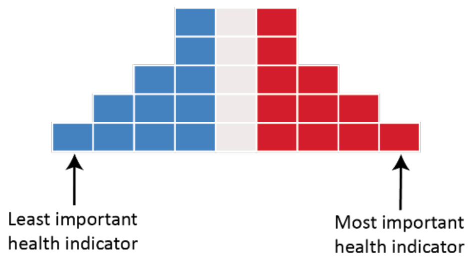

We are most interested in your personal opinions and there are no right or wrong answers.

Here is a brief video (2.5 minutes) that explains in general terms how to undertake this exercise. https://youtu.be/zgT2N4zcPtQ

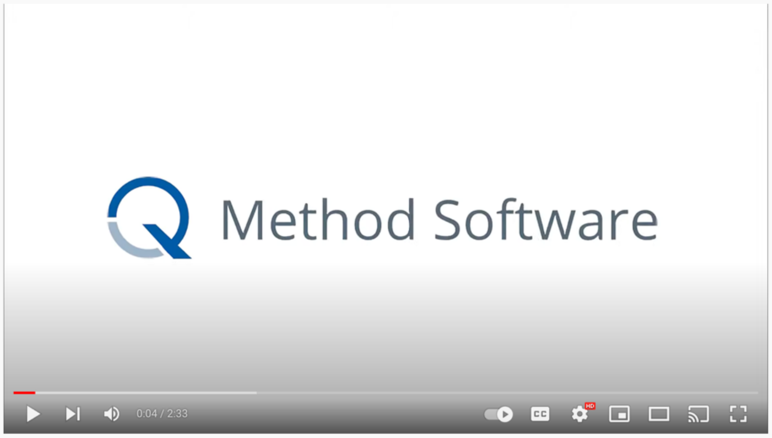

Participant Tutorial: How to Complete a Q-Sort

We are also interested in why you might choose one statement over another so there will be a brief survey at the end of the sorting exercise. You may skip this final survey if you wish.

Additional Information for Participants (provided during the q-sort exercise)

All of these outcomes/health indicators that have been chosen are considered important by some or all of the people we have spoken to when preparing this survey. What we are most interested is however is which outcomes/health indicators you think (from your own personal experiences) rank higher or lower compared to the others on the list.

You should aim to place the outcomes/health indicators that you think are the most important to the right of the pyramid. Conversely the outcomes/health indicators that you think are relatively less important when compared to the other outcomes/health indicators in this list should be placed to the left of the pyramid. Most indicators will be in the middle of the pyramid. Here is a short video (2.5 minutes explaining how to undertake this exercise) https://youtu.be/zgT2N4zcPtQ

There are no right or wrong answers!

There is no time limit to this exercise.

If you are having difficulty with the exercise and want further advice, explanations, or hard copies of the list you can email the researcher at candersen@georgeinstitute.org.au

You can press the reset button to start again at any time.

Pre Q-Sort Questions

1. What is your age? (multiple choice)

- <26
- 26-35
- 36-45
- 46-55
- 56-65
- >65

2. What is your gender? (multiple choice)

- Man
- Woman
- Transgender
- Non-binary
- Prefer not to say
- Let me type… [opens free text option]

3. With respect to subarachnoid haemorrhage would you describe yourself as? (multiple choice)

- Survivor, family member and/or caregiver
- A health care provider (with only limited research involvement)
- Someone involved in research, medical publishing, policy making or industry

3a. For survivors, family members and/or caregivers
Are you a? (multiple choice)

- Survivor
- Family member and/or caregiver

3b. For health care providers and researchers
How would you describe your health care role or previous training? (multiple choice)

- Researcher not previously trained in a health care role
- Nurse
- Interventional or Diagnostic Radiologist
- Neurosurgeon
- Neurologist or Stroke Physician
- Rehabilitation doctor
- Speech Therapist
- Anaesthetist or anaesthesiologist
- Intensive Care Doctor

- Family or General Practice Physician
- Occupational Therapist
- Free text option to self describe

4. How long have you had an association with SAH as either a patient/family/caregiver, health care professional or researcher? (multiple choice)

- Less than 12 months
- 1-2 years
- 2-5 years
- >5 years

5. What geographical region do you come from? (multiple choice)

- Caribbean, Central or North America
- Asia
- Africa
- Oceania
- Europe
- South America

6. What is your country of residence? (free text)

Post Sort Questions

1. Can you explain in your own words what you think are the most important outcomes or health indicators after SAH that we should measure when evaluating new treatments and why? (free text)

2. In your opinion were there any outcomes, domains or health indicators that were missing from the sorting list and should be considered? (free text)

3. How did you find the sorting exercise? (multiple choice)

- Straightforward
- Challenging but achievable
- Too confusing

4. The q-sort is a novel type of survey, would you like to provide some feedback on your experience and/or any comments more generally? (free text)

Appendix 3 – Factor rankings and representative q-sorts

Factor 1 – Representative Q-Sort

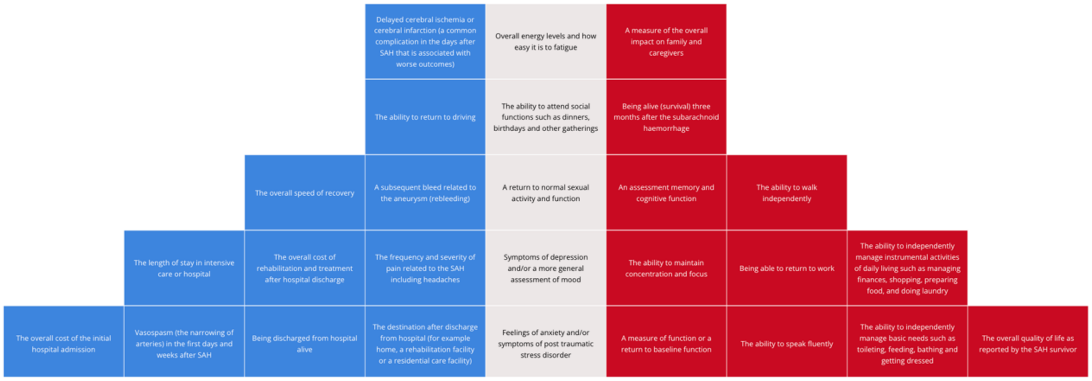

Factor 1 Rankings (most important to least important)

26	The overall quality of life as reported by the SAH survivor
13	The ability to independently manage basic needs such as toileting, feeding, bathing and getting dressed
14	The ability to independently manage instrumental activities of daily living such as managing finances, shopping, preparing food, and doing laundry
7	The ability to speak fluently
15	Being able to return to work
19	The ability to walk independently
18	A measure of function or a return to baseline function
3	The ability to maintain concentration and focus
27	An assessment memory and cognitive function
25	Being alive (survival) three months after the subarachnoid haemorrhage
16	A measure of the overall impact on family and caregivers
1	Feelings of anxiety and/or symptoms of post traumatic stress disorder
2	Symptoms of depression and/or a more general assessment of mood
6	A return to normal sexual activity and function
12	The ability to attend social functions such as dinners, birthdays and other gatherings
4	Overall energy levels and how easy it is to fatigue
20	The destination after discharge from hospital (for example home, a rehabilitation facility or a residential care facility)
5	The frequency and severity of pain related to the SAH including headaches
11	A subsequent bleed related to the aneurysm (rebleeding)
17	The ability to return to driving
8	Delayed cerebral ischemia or cerebral infarction (a common complication in the days after SAH that is associated with worse outcomes)
24	Being discharged from hospital alive
22	The overall cost of rehabilitation and treatment after hospital discharge
10	The overall speed of recovery
9	Vasospasm (the narrowing of arteries) in the first days and weeks after SAH
23	The length of stay in intensive care or hospital
21	The overall cost of the initial hospital admission

Factor 2 – Representative Q-Sort

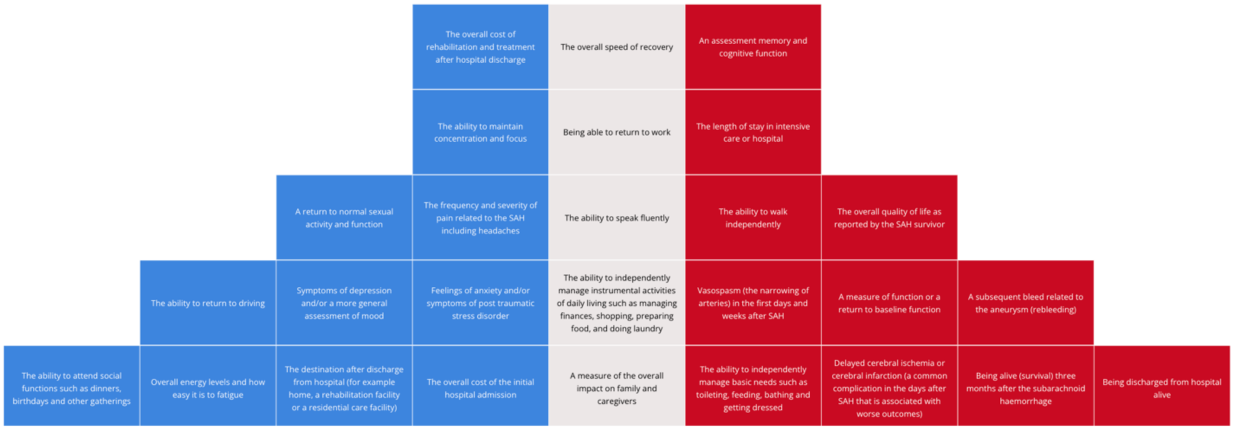

Factor 2 Rankings (most important to least important)

24	Being discharged from hospital alive
25	Being alive (survival) three months after the subarachnoid haemorrhage
11	A subsequent bleed related to the aneurysm (rebleeding)
8	Delayed cerebral ischemia or cerebral infarction (a common complication in the days after SAH that is associated with worse outcomes)
18	A measure of function or a return to baseline function
26	The overall quality of life as reported by the SAH survivor
13	The ability to independently manage basic needs such as toileting, feeding, bathing and getting dressed
9	Vasospasm (the narrowing of arteries) in the first days and weeks after SAH
19	The ability to walk independently
23	The length of stay in intensive care or hospital
27	An assessment memory and cognitive function
16	A measure of the overall impact on family and caregivers
14	The ability to independently manage instrumental activities of daily living such as managing finances, shopping, preparing food, and doing laundry
7	The ability to speak fluently
15	Being able to return to work
10	The overall speed of recovery
21	The overall cost of the initial hospital admission
1	Feelings of anxiety and/or symptoms of post traumatic stress disorder
5	The frequency and severity of pain related to the SAH including headaches
3	The ability to maintain concentration and focus
22	The overall cost of rehabilitation and treatment after hospital discharge
20	The destination after discharge from hospital (for example home, a rehabilitation facility or a residential care facility)
2	Symptoms of depression and/or a more general assessment of mood
6	A return to normal sexual activity and function
4	Overall energy levels and how easy it is to fatigue
17	The ability to return to driving
12	The ability to attend social functions such as dinners, birthdays and other gatherings

Factor 3– Representative Q-Sort

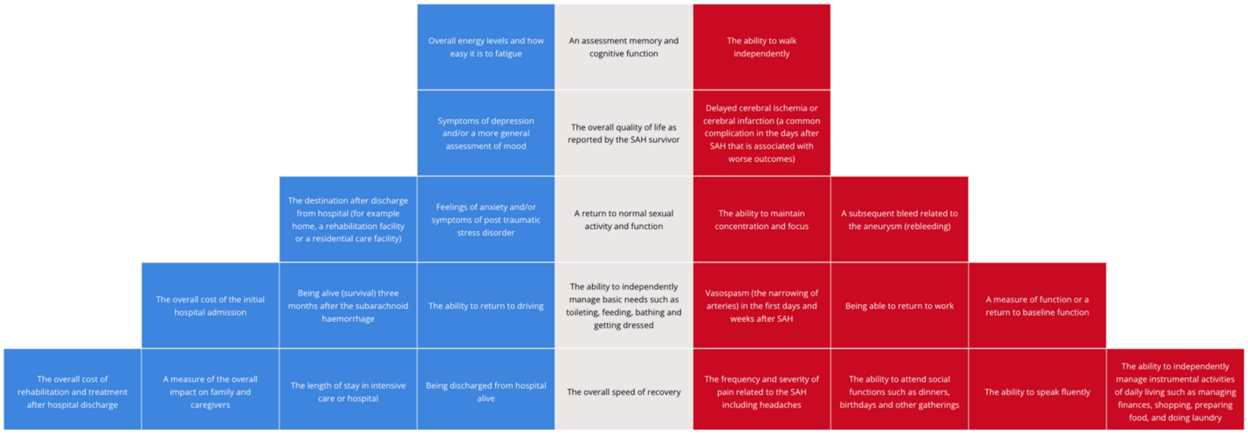

Factor 3 Rankings (most important to least important)

14	The ability to independently manage instrumental activities of daily living such as managing finances, shopping, preparing food, and doing laundry
7	The ability to speak fluently
18	A measure of function or a return to baseline function
12	The ability to attend social functions such as dinners, birthdays and other gatherings
15	Being able to return to work
11	A subsequent bleed related to the aneurysm (rebleeding)
5	The frequency and severity of pain related to the SAH including headaches
9	Vasospasm (the narrowing of arteries) in the first days and weeks after SAH
3	The ability to maintain concentration and focus
8	Delayed cerebral ischemia or cerebral infarction (a common complication in the days after SAH that is associated with worse outcomes)
19	The ability to walk independently
10	The overall speed of recovery
13	The ability to independently manage basic needs such as toileting, feeding, bathing and getting dressed
6	A return to normal sexual activity and function
26	The overall quality of life as reported by the SAH survivor
27	An assessment memory and cognitive function
24	Being discharged from hospital alive
17	The ability to return to driving
1	Feelings of anxiety and/or symptoms of post traumatic stress disorder
2	Symptoms of depression and/or a more general assessment of mood
4	Overall energy levels and how easy it is to fatigue
23	The length of stay in intensive care or hospital
25	Being alive (survival) three months after the subarachnoid haemorrhage
20	The destination after discharge from hospital (for example home, a rehabilitation facility or a residential care facility)
16	A measure of the overall impact on family and caregivers
21	The overall cost of the initial hospital admission
22	The overall cost of rehabilitation and treatment after hospital discharge

Factor 4– Representative Q-Sort

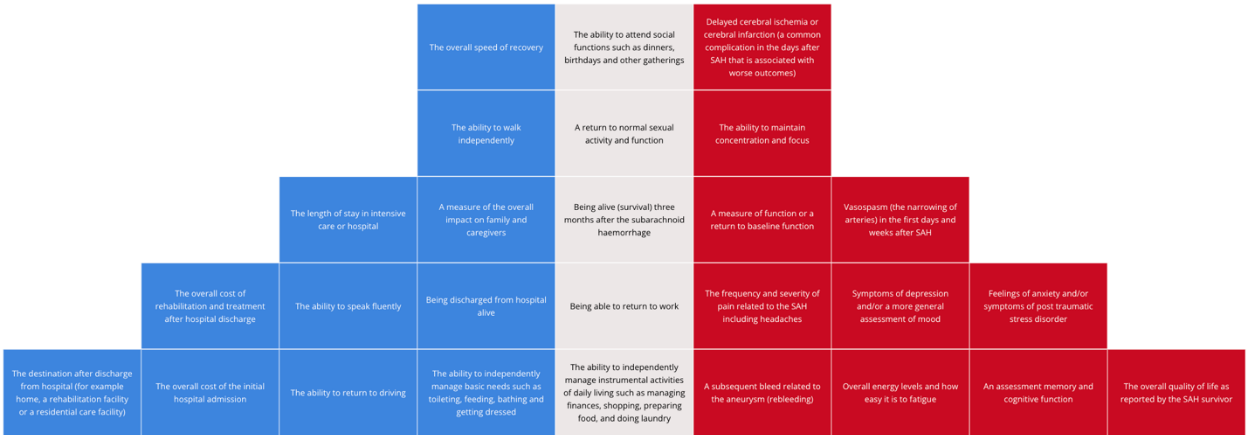

Factor 4 Rankings (most important to least important)

26	The overall quality of life as reported by the SAH survivor
27	An assessment memory and cognitive function
1	Feelings of anxiety and/or symptoms of post traumatic stress disorder
4	Overall energy levels and how easy it is to fatigue
2	Symptoms of depression and/or a more general assessment of mood
9	Vasospasm (the narrowing of arteries) in the first days and weeks after SAH
11	A subsequent bleed related to the aneurysm (rebleeding)
5	The frequency and severity of pain related to the SAH including headaches
18	A measure of function or a return to baseline function
3	The ability to maintain concentration and focus
8	Delayed cerebral ischemia or cerebral infarction (a common complication in the days after SAH that is associated with worse outcomes)
14	The ability to independently manage instrumental activities of daily living such as managing finances, shopping, preparing food, and doing laundry
15	Being able to return to work
25	Being alive (survival) three months after the subarachnoid haemorrhage
6	A return to normal sexual activity and function
12	The ability to attend social functions such as dinners, birthdays and other gatherings
13	The ability to independently manage basic needs such as toileting, feeding, bathing and getting dressed
24	Being discharged from hospital alive
16	A measure of the overall impact on family and caregivers
19	The ability to walk independently
10	The overall speed of recovery
17	The ability to return to driving
7	The ability to speak fluently
23	The length of stay in intensive care or hospital
21	The overall cost of the initial hospital admission
22	The overall cost of rehabilitation and treatment after hospital discharge
20	The destination after discharge from hospital (for example home, a rehabilitation facility or a residential care facility)

Factor 5– Representative Q-Sort

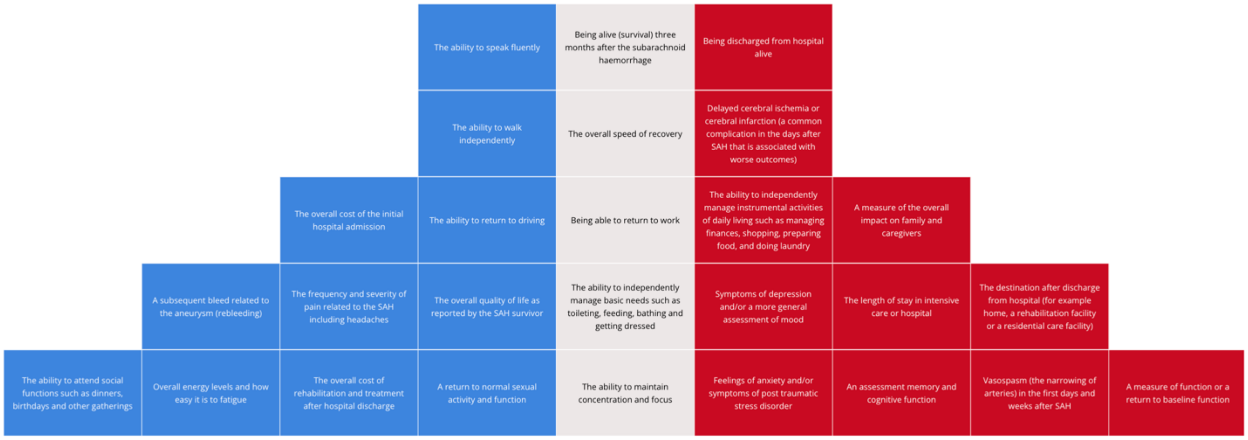

Factor 5 Rankings (most important to least important)

18	A measure of function or a return to baseline function
9	Vasospasm (the narrowing of arteries) in the first days and weeks after SAH
20	The destination after discharge from hospital (for example home, a rehabilitation facility or a residential care facility)
27	An assessment memory and cognitive function
23	The length of stay in intensive care or hospital
16	A measure of the overall impact on family and caregivers
1	Feelings of anxiety and/or symptoms of post traumatic stress disorder
2	Symptoms of depression and/or a more general assessment of mood
14	The ability to independently manage instrumental activities of daily living such as managing finances, shopping, preparing food, and doing laundry
8	Delayed cerebral ischemia or cerebral infarction (a common complication in the days after SAH that is associated with worse outcomes)
24	Being discharged from hospital alive
3	The ability to maintain concentration and focus
13	The ability to independently manage basic needs such as toileting, feeding, bathing and getting dressed
15	Being able to return to work
10	The overall speed of recovery
25	Being alive (survival) three months after the subarachnoid haemorrhage
6	A return to normal sexual activity and function
26	The overall quality of life as reported by the SAH survivor
17	The ability to return to driving
19	The ability to walk independently
7	The ability to speak fluently
22	The overall cost of rehabilitation and treatment after hospital discharge
5	The frequency and severity of pain related to the SAH including headaches
21	The overall cost of the initial hospital admission
4	Overall energy levels and how easy it is to fatigue
11	A subsequent bleed related to the aneurysm (rebleeding)
12	The ability to attend social functions such as dinners, birthdays and other gatherings
